# *Suttonella ornithocola* detected within lesions of tit birds (*Paridae*) from epidemic death episodes in Germany, 2018–2020

**DOI:** 10.3389/fvets.2022.977570

**Published:** 2022-09-08

**Authors:** Eva Leitzen, Martin Peters, Sabine Merbach, Peter Wohlsein, Wolfgang Baumgärtner

**Affiliations:** ^1^Department of Pathology, University of Veterinary Medicine Hannover Foundation, Hannover, Germany; ^2^Chemical and Veterinary Investigation Office Westphalia, Arnsberg, Germany

**Keywords:** *Suttonella ornithocola*, pneumonia, tit (*Paridae*), *in situ*, avian

## Abstract

Several episodes of increased mortality in wild birds of the families *Paridae* and *Aegithalidae* have been documented in recent decades. The majority of affected animals exhibited necrotizing pneumonia with intralesional bacteria. *Suttonella* (*S*.) *ornithocola*, a gram-negative bacterium in the *Cardiobacteriaceae* family, has been regularly cultured bacteriologically from affected birds and has long been suspected as a potentially fatal cause of respiratory disease in birds. However, a direct causal relationship between this specific bacterium and the observed lesions within birds has not yet been established. Therefore, postmortem tissue from six tits was used in the present study, including three blue tits (*Cyanistes caeruleus*) and three great tits (*Parus major*). Five of the six tits tested positive for *S. ornithocola* in bacteriological examination and originated from two incidents of increased mortality in *Paridae* in Germany. Animals found dead in the administrative district of Arnsberg (North Rhine Westphalia) in 2018 and 2020 were investigated for genomic fragments of *S. ornithocola* by chromogenic *in situ* hybridization using a newly developed DNA probe based on publicly assessable DNA sequences of the 16S rRNA gene of *S. ornithocola*. Positive hybridization signals were detected in five out of five animals and were predominantly detected within necrotizing lesions in lung and occasionally in lesions affecting liver and trachea. Interestingly, the lung of one animal without obvious necrotizing pulmonary lesions revealed positive hybridization results in the lumen of one pulmonary blood vessel. Two negative controls, including one bacteriologically *S. ornithocola*-negative great tit and a cattle egret (*Bubulcus ibis*) suffering from salmonellosis, did not yield positive signals, indicating high sensitivity and specificity of the probe used. This is the first time that *S. ornithocola* has been clearly identified within necrotizing lesions in deceased tits. Although Koch's postulates have yet to be fulfilled, positive hybridization signals in association with detectable lesions are considered as further and strong evidence of the significant contribution of *S. ornithocola* to the several episodes of tit mortality recorded in Germany.

## Introduction

During spring of 2020 an increased mortality of singing birds, especially of European blue tits (*Cyanistes caeruleus*) and to a lesser extent also great (*Parus major*) and coal tits (*Parus ater*), was observed in North Rhine Westphalia, Germany (1). This commemorated a series of fatalities in tits (*Paridae*) during spring 2018, where diseased and dead tits were observed in multiple gardens with feeding stations in southern North Rhine-Westphalia (2). Postmortem investigation frequently revealed pulmonary congestion and pneumonia as well as discolored intestinal content with enteritis in affected birds (1, 2). In both events, *Suttonella* (*S*.) *ornithocola* was frequently detected in organ samples from deceased birds via microbiological diagnosis, polymerase chain reaction (PCR) and DNA sequence analysis of the 16S rRNA gene. Therefore, it was suspected to be a potential cause or comorbidity leading to increased tit mortality in Germany.

*S. ornithocola* was first isolated from congested lungs of deceased birds in England and Wales during 1995/1996 and was suggested as possible cause for the widespread death of predominantly tit species within the United Kingdom. It is a fastidious ß-hemolytic and gram-negative coccobacillus preferring microaerophilic culture conditions, that can represent a challenge in cultivation (3, 4). Today, molecular techniques such as polymerase chain reaction and sequence analysis are widely used for the precise and reliable detection of this pathogen (4). It is postulated to target the upper and lower respiratory tract of predominantly *Paridae* and is suspected to cause a potentially fatal disease (3–5). Bacteria of the genus *Suttonella* belong to the family *Cardiobacteriaceae* and are closely related to members of the genera *Dichelobacter* and *Cardiobacterium* (6). Members of these genera are known to contribute to diseases in animals, including contagious footrot in cloven-hoofed animals (7, 8). In humans, they have been identified in association with endocarditis (9–11). In birds, *S. ornithocola* has been isolated from small intestine, liver, spleen, kidney, heart and blood in addition to lung tissue, indicating hematogenic spread, but has rarely been associated with macroscopic or histologic lesions other than congestion and necrotizing pneumonia (1–3, 5). However, evidence of direct causality between the isolation of the bacterium from dead or diseased animals and the development of lesions is lacking.

*In situ* hybridization combines the spatial detection of potential pathogens within tissues and lesion sites with the possibility to clearly identify and detect microorganisms using specific microbial DNA and/or RNA sequences comparable to microbial fingerprints (12–14). Therefore, a specific *in situ* probe targeting 50 nucleotides in length of the 16S rRNA gene was designed to detect *S. ornithocola* within formalin-fixed, paraffin-embedded (FFPE) tissue of affected birds. Chromogenic *in situ* hybridization (CISH) using a digoxigenin-labeled DNA-probe was performed. To the author's knowledge, this is the first visualization of specific genomic fragments of *S. ornithocola* within characteristic lesions in deceased birds.

## Materials and methods

### Animals and tissues

The present study includes six male tits (*Paridae*), including three blue tits (*Cyanistes caeruleus*) and three great tits (*Parus major*), which were found dead in the administrative district of Arnsberg, North Rhine Westphalia in Germany during the incidents in 2018 (case 1) and 2020 (cases 2-6) (1, 2). Five of six tits (cases 1–5) tested positive for *S. ornithocola* in bacteriology. Moreover, one archived case of a male, captive held cattle egret (*Bubulcus ibis*), found moribund in its enclosure in a German zoo in 2015 that died from confirmed salmonellosis (case 7) was added (Table 1). All animals underwent routine necropsy and representative organ samples (Table 2) were harvested for histological tissue processing with special emphasis on central nervous system, lung, liver, spleen, and gastrointestinal system. Moreover, further analyses using microbiological, virological, and parasitological testing (Table 1) were conducted. Positive results of the microbiological examination were validated by PCR. PCR analysis was performed according to an in-house protocol of the Friedrich-Loeffler-Institut in Jena, Germany. DNA sequencing of the 16S rRNA gene was performed as previously described (1, 2).

**Table 1:** Overview of the investigated animals, summarizing the results of the microbiological, parasitological, and molecular examinations carried out on the cases examined.

**Case No**	**Species**	**Preservation**	**Microbiology**	**Parasitology (flotation method)**	**16S rRNA gene sequencing**	**PCR**
1	Great tit	Unknown	• Lung: +++ *S. ornithocola*; +++ *E. faecalis* • Liver: +++ *S. ornithocola*; ++ coliform bacteria • Small intestine: ++ coliform bacteria	Negative	Suttonella +	*Suttonella* pos. (isolate)
2	Great tit	Unknown	• Lung: +++ *S. ornithocola* • Liver: +++ *S. ornithocola* • Small intestine: +++ coliform bacteria	Tapeworms	Not performed	*Suttonella* pos. (pooled sample of liver, lung, heart, kidney, intestine)
3	Blue tit	Moderate autolysis	• Lung: + *S. ornithocola* • Liver: ++ *S. ornithocola* • Spleen: – • Small intestine: + coliform bacteria	Negative	Not performed	*Suttonella* pos. (isolate)
4	Blue tit	Unknown	• Lung: +++ *S. ornithocola* • Liver: +++ *S. ornithocola* • Small intestine: ++ alpha-hem. streptococci	Negative	Not performed	*Suttonella* pos. (pooled sample of liver, lung, heart, spleen, kidney, central nervous system)
5	Blue tit	Unknown	• Lung: +++ *S. ornithocola* • Liver: +++ *S. ornithocola* • Small intestine: +++ coliform bacteria	Negative	Not performed	*Suttonella* pos. (intestine)
6	Great tit	Unknown	• Lung: – • Liver: – • Small intestine: ++ *Enterococcus* spp.	Negative	Not performed	Not performed
7	Cattle egret	Mild autolysis	• Liver: *Salmonella* ser. Typhimurium	Not performed	Not performed	Not performed

S., Suttonella; –, negative result; +, isolated in small numbers; ++, isolated in moderate numbers; +++, isolated in large numbers; pos., positive PCR result.

**Table 2:** Overview of the investigated animals summarizing the corresponding results of the histological and *in situ* hybridization analyses carried out on the cases examined.

**Case No**	**Organs**	**Histologic lesions**	**Positive *in situ* hybridization signals**
1	Lung	Mf., mild to moderate, necrotizing pneumonia with intralesional bacteria	mf., mild
	Liver	Hyperemia; fo. granulomatous infiltrate	NHS
	Heart	NSML	NHS
	CNS	NSML	NHS
	Ventriculus	NSML	NHS
	Testis	NSML	NHS
	Kidney	NSML	NHS
	Skin	Fo., mild pustular dermatitis	NHS
2	Lung	Mf., moderate to severe, necrotizing pneumonia with intralesional bacteria	mf., moderate
	Liver	NSML	NHS
	Heart	Intravascular bacteria	NHS
	CNS	NSML	NHS
	Pro-ventriculus	NSML	NHS
	Ventriculus	NSML	NHS
	Intestine	Tapeworms	NHS
	Pancreas	NSML	NHS
	Testis	NSML	NHS
	Kidney	NSML	NHS
	Spleen	Follicular hyperplasia	NHS
3	Lung	Hyperemia, edema, mixed cellular infiltrates, intravascular bacterial structures	fo., mild, intravascular
	Liver	NSML	NHS
	Heart	NSML	NHS
	CNS	NSML	NHS
	Ventriculus	NSML	NHS
	Testis	NSML	NHS
4	Lung	Hyperemia, edema	fo., inconclusive
	Trachea	Necrotizing inflammation with intralesional bacteria	fo., severe
	Liver	Hyperemia	NHS
	Heart	NSML	NHS
	CNS	NSML	NHS
	Ventriculus	NSML	NHS
5	Lung	mf., moderate, necrotizing pneumonia with intralesional bacteria	mf., moderate
	Liver	mf., necroses with intralesional bacterial structures	mf., mild
	Heart	NSML	NHS
	CNS	NSML	NHS
	Kidney	NSML	NHS
	Bone	NSML	NHS
	Skeletal muscle	NSML	NHS
6	Lung	Hyperemia	NHS
	Heart	NSML	NHS
	liver	Hyperemia	NHS
	CNS	NSML	NHS
	Pro-ventriculus	NSML	NHS
	Ventriculus	NSML	NHS
	Small intestine	NSML	NHS
	Pancreas	NSML	NHS
	Testis	NSML	NHS
	Kidney	Mf., interstitial, lymphocytic infiltration	NHS
	Spleen	Follicular hyperplasia	NHS
	Bone	NSML	NHS
	Skeletal muscle	Mf., lympho-histiocytic myositis	NHS
	Feathered skin	NSML	NHS
7	Lung	Hyperemia; multiple bacterial emboli; fo., mild, granulomatous inflammation;	NHS
	Liver	mf., heterophilic and necrotizing inflammation with bacterial colonies; mf., severe, fibrinous-heterophilic to necrotizing, partly granulomatous inflammation;	NHS
	CNS	NSML	NHS
	Spleen	Multiple bacterial emboli	NHS

CNS, central nervous system; NSML, no significant microscopic lesions; NHS, no hybridization signals; fo., focal; mf., multifocal.

### Probe design and *in situ*-hybridization

Probe design was carried out by screening the genome of *S. ornithocola* using DDBJ/EMBL/GenBank and Primer-BLAST for identification of suitable sequences for high hybridization specificity and suitable GC content (15). For detection of *Suttonella*-specific sequences, a DNA-probe was designed. A 50 nucleotide *in situ* probe targeting positions 431–481 of the 16S rRNA gene of *S. ornithocola* (type strain B6/99/2; GenBank: AJ717394.1) was selected and commercially produced (Eurofins Genomics GmbH, Ebersberg, Germany). CISH was performed using FFPE tissue samples screening a representative organ spectrum of all seven birds. The infection status of tits was verified by bacteriological culture in all animals with MALDI-TOF identification of *S. ornithocola*. Positive bacteriological findings (cases 1–5) were validated by PCR and confirmed by sequence analysis for one case (case 1) (1, 2). Appropriate tissue samples of one non-infected tit (case 6) as well as from one cattle egret (case 7) suffering from a confirmed infection with *Salmonella* ser. Typhimurium served as negative controls. Sections of FFPE material were cut at 2–3 μm thickness on a microtome and mounted on SuperFrost-Plus® slides (Thermo Fisher Scientific Inc., Fisher Scientific GmbH). Hybridization was carried out as described before (14, 16–19). Briefly, sections were dewaxed and hydrated in Roti-Histol (Carl Roth) followed by a series of graded ethanol and subsequent washing using diethylpyrocarbonate-treated water. Proteolytic digestion was achieved using 1 μg/ml of proteinase K (Roche Diagnostics) followed by post-fixation, acetylation, pre-hybridization and DNA-denaturation (10 min, 99°C). Afterwards, hybridization (overnight, 52°C) with a probe concentration of 500 ng/ml was performed. For visualization of successful probe-binding, an alkaline phosphatase-labeled anti-digoxigenine-antibody (1:200; Roche Diagnostics) in combination with nitro blue tetrazolium chloride (NBT; Sigma-Aldrich Chemie, Taufkirchen, Germany) and 5-bromo-4-chloro-3-indolyl phosphate (BCIP, X-Phosphate; Sigma-Aldrich Chemie) as substrates was applied. Positive signals presented as purple precipitates in the affected tissues. For negative controls, tissue was incubated with hybridization buffer only. Pictures of representative areas were taken using a DP72 camera (Olympus) mounted on a BX51 microscope (Olympus). For *in situ* hybridization pictures, differential interference contrast microscopy (Nomarski microscopy) was used. High magnification insets were taken using the Olympus SLIDEVIEW VS200 slide scanner with a 40 × oil objective and digital zoom.

## Results

### Clinical history and gross necropsy findings

All seven birds included within the study were male and found moribund or dead during spring. Fluffed feathers, inertness, disorientation, and lack of responsiveness were observed in some of the affected birds. All carcasses were completely available for pathological examination. Birds showed variable states of preservation and nutrition. Tits frequently had moist and/or red-black marbled lungs (*n* = 4; cases 1, 2, 3, 4) and reddish to black intestinal contents (*n* = 4; cases 1, 2, 4, 5). Moreover, deceased tits partly exhibited enlarged spleen (*n* = 2; cases 1 and 6) and liver (*n* = 1; case 6), infestation with intestinal tapeworms (*n* = 1; case 2) as well as blunt trauma to the skull along with multifocal hemorrhages within the coelomic cavity (*n* = 1; case 5). The cattle egret (case 7) showed multifocal white, pinhead-sized lesions on lung, liver and spleen as well as a reddish discoloration of the cloaca.

### Microscopic findings

**Figure 1:**
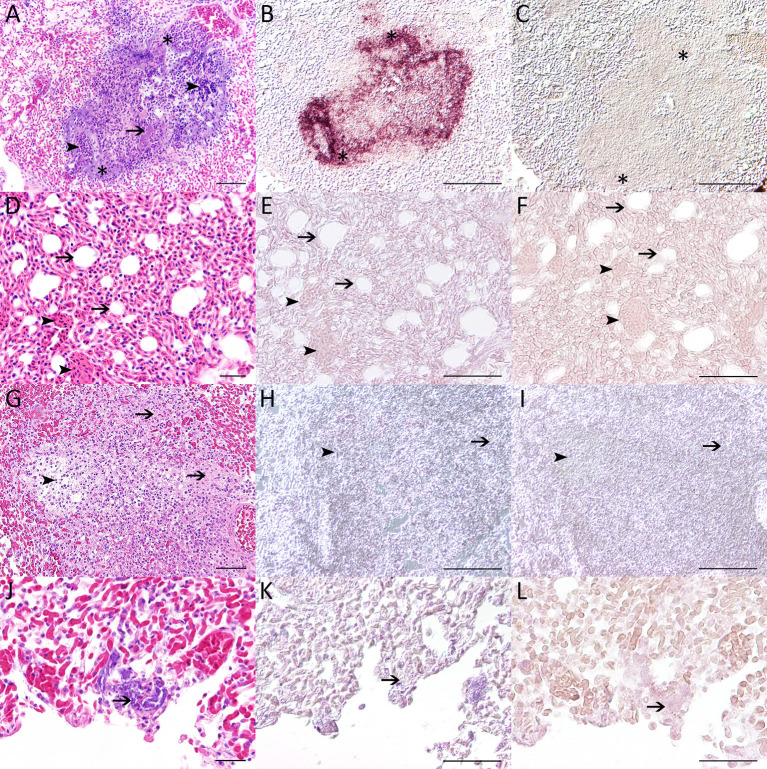
Pictures show hematoxylin and eosin stain **(A,D,G,J)**, *in situ* hybridization using the anti-*Suttonella in situ* probe **(B,E,H,K)** and results after incubation with hybridization buffer only **(C,F,I,L)**. Pulmonary lesions within an affected tit [case 2; **(A–C)**] characterized by severe loss of organotypical architecture with accumulation of cellular debris (arrow) and inflammatory cells (arrowheads) as well as intralesional bacteria (asterisks). Necrotizing lesions with intralesional bacteria within the lung tissue of the affected animal show a strong positive reaction (purple precipitate; asterisks) in *in situ* hybridization **(B)**. Lung tissue of the negative control tit [case 6; **(D–F)**] consisting of regularly structured pulmonary tissue with air capillaries (arrows) and blood vessels (arrowheads). Pulmonary lesion of the cattle egret (case 7) suffering from salmonellosis displaying a focus of severe, fibrino-necrotizing pneumonia **(G–I)** with cellular deposition of debris and fibrinous material (arrow), inflammatory cells (arrowheads) and multifocal bacterial emboli [arrow; **(J–L)**]. No hybridization signal was detected within the lung of the negative control tit [**(E)**; air capillaries: arrows; blood vessels: arrowheads] and the pulmonary lesions of the cattle egret [**(H)**; debris and fibrinous material: arrow; inflammatory cells: arrowhead]. In addition, no signals were detected without using the DNA probe but hybridization buffer only **(C,F,I)**—the respective areas of interest are correspondingly marked with asterisks, arrows and arrowheads **(C,F,I)**. Pictures displaying *in situ* hybridization were taken using differential contrast (Nomarski) microscopy. Bars: 20 μm: **(D)**; 50 μm: **(A,E–G)**; 100 μm: **(B,C,H,I)**.

**Figure 2:**
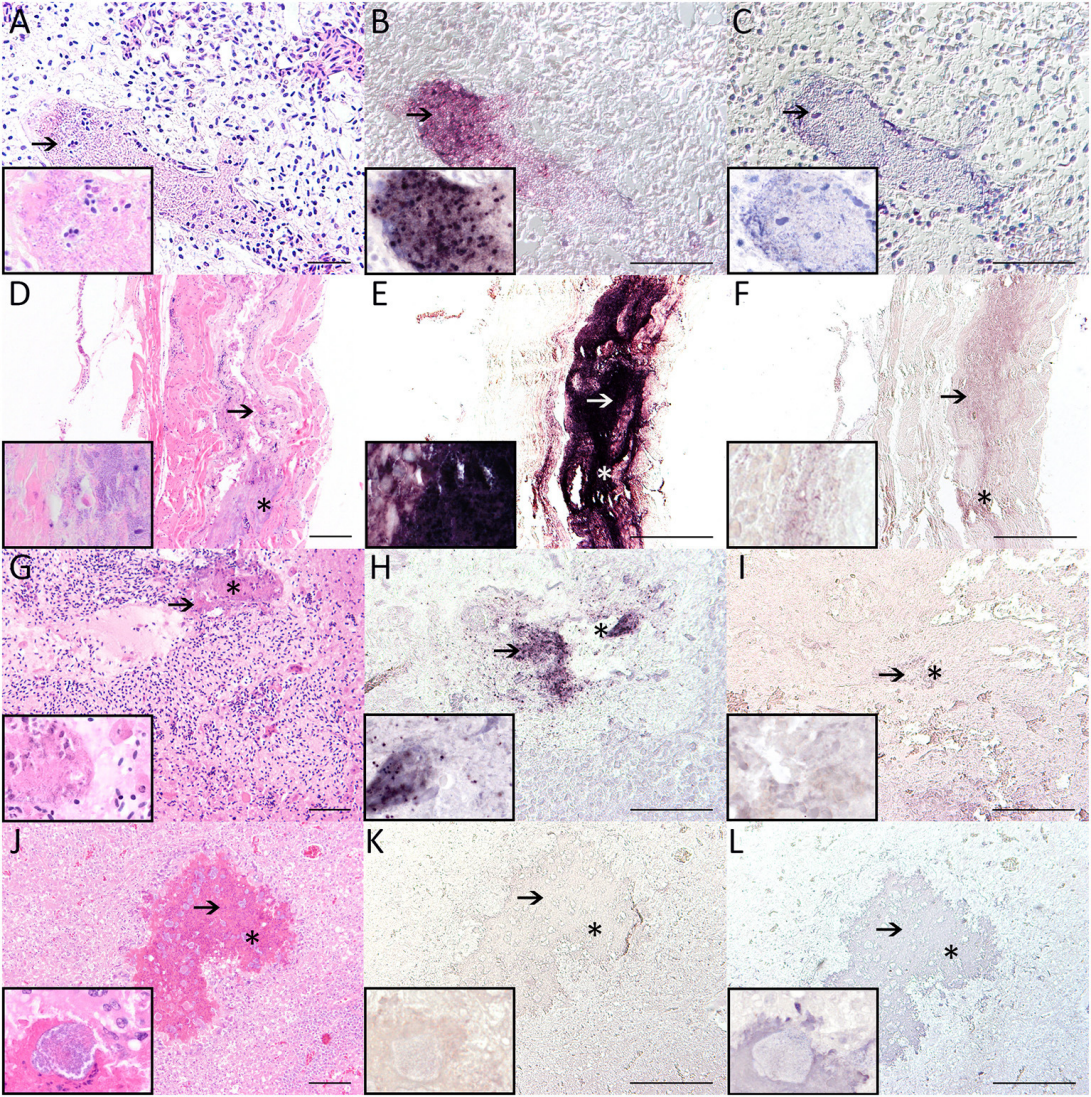
Pictures show hematoxylin and eosin stain **(A,D,G,J)**, *in situ* hybridization using the anti-*Suttonella in situ* probe **(B,E,H,K)** and results after incubation with hybridization buffer only **(C,F,I,L)**. Pulmonary vessel (case 3) with intraluminal bacteria [arrow; **(A–C)**] as well as observed tracheal [case 4; **(D–F)**] and hepatic lesions [case 5; **(G–I)**], which are characterized by loss of organotypical architecture, accumulation of debris (arrows) and presence of bacterial structures (asterisks), show a strong positive reaction (purple precipitate; arrows) in *in situ* hybridization **(B,E,H)**. Hepatic lesions of the cattle egret (case 7) suffering from salmonellosis **(J,K)** also show loss of regular organ architecture, cellular debris (arrows), and bacterial colonies (asterisks). No signal was detected within hepatic lesions of the cattle egret [**(K)** bacteria: asterisks]. In addition, no signals were detected without using the DNA probe but hybridization buffer only **(C,F,I,L)**—the respective areas of interest are correspondingly marked with asterisks and arrows. Insets detail bacteria in high magnification (100 × ). Pictures displaying *in situ* hybridization were taken using differential contrast (Nomarski) microscopy. Bars: 20 μm: **(A)**; 50 μm: **(B,C,G)**; 100 μm: **(D,H,I,J)**; 200 μm: **(E,F,K,L)**.

Histological examination (Table 2) revealed necrotizing pneumonia with intralesional rod-shaped, gram-negative (Supplementary Figure 1A) bacterial structures in three birds (cases 1, 2, 5; Figure 1A). Pulmonary lesions were characterized by loss of organotypic architecture and loss of cellular details (necrosis), hemorrhage and infiltration of macrophages and heterophils. One (case 3) exhibited mixed cellular infiltrates and edema of the lung, and three (cases 3, 4, 6) hyperemia. One affected bird (case 4) displayed a focally extensive lesion within the trachea characterized by loss of organotypic architecture and cellular details (necrosis) with high numbers of intralesional bacteria, acutely swollen myocytes and infiltration of low numbers of macrophages and heterophils (Figure 2D). In addition, evidence of bacterial colonization associated with liver necrosis was found in one bird (case 5; Figure 2G). Again, the lesion was characterized by loss of organotypic architecture and cellular details (necrosis) and was accompanied by hemorrhage. Detached, hypereosinophilic, and occasionally karyorrhectic hepatocytes were noted in the periphery of the lesion. Within another case (case 2), gram-negative, coccoid bacterial emboli were found in the lumen of a cardiac vessel (Supplementary Figure 1B). One bird (case 3) exhibited intravascular accumulation of bacterial structures within a pulmonary vessel (Figure 2A). Three birds (cases 1, 4, 6) had hyperemia of liver, one (case 1) had a focal granulomatous infiltrate within liver tissue, and two (cases 2, 6) displayed lymphoid hyperplasia of the spleen. In one bird (case 6) a lymphocytic nephritis, as well as a multifocal lympho-histiocytic myositis were found. One tit (case 1) had a focal pustular dermatitis. Diffuse hyperemia, fibrino-necrotizing, and granulomatous pneumonia as well as multiple gram-negative bacterial emboli within lung (Figures 1G–L) and liver (Supplementary Figure 1C) were observed in the cattle egret (case 7). Moreover, a heterophilic to necrotizing and partly granulomatous hepatitis with intralesional bacteria as well as a heterophilic to histiocytic myocarditis and a heterophilic to necrotizing and granulomatous inflammation of the cloaca were detected within this animal. Bacterial emboli were also present within the spleen of this bird.

### Further investigations

In five of seven birds (cases 1–5) *S. ornithocola* was detected in lung and liver during bacteriological examination (Table 1). Coliform bacteria were cultured from liver (case 1) and frequently from small intestine (cases 1–3, 5). Moreover, *Enterococcus faecalis* was detected in lung tissue of one bird (case 1). *Enterococcus* spp. (case 6) and alpha-hemolytic Streptococci (case 4) were occasionally cultured from small intestine. In one case (case 1), 16S rRNA sequencing of lung tissue was performed to prove presence of *S. ornithocola*. In five cases (cases 1–5), infection with *S. ornithocola* was confirmed by MALDI-TOF and PCR using a representative organ spectrum. Bacteriological examination of one great tit (case 6; negative control) with unremarkable lung findings on macroscopic and histologic examination revealed no evidence of infection with *S. ornithocola*. *Salmonella* ser. Typhimurium was cultured from liver samples of the cattle egret (case 7). All birds (cases 1–7) tested negative for avian influenza. Five animals (cases 2–6) tested negative for Usutu virus and West Nile virus. One animal (case 2) tested positive for tapeworms during parasitological examination using flotation method.

### *In situ* hybridization

All three cases exhibiting necrotizing pneumonia (cases 1, 2, 5) showed positive hybridization signals (Table 2) within affected lung tissue (Figures 1B,C). Strong, specific signals were detected using the antisense probe followed by antibody-alkaline phosphatase detection. No signals were detected after hybridization with the sense probe (Supplementary Figure 2). In one (case 3) of the two cases that tested positive for *S. ornithocola* on bacteriological examination but had no evidence of necrotizing pneumonia, only pulmonary edema and foci of punctate positive signal were detected in the lumen of a pulmonary vessel (Figures 2B,C). The second animal (case 4) showed only a focal, inconclusive hybridization signal in the lung, but a focal, strong signal within the trachea (Figures 2D–F). Within one bird (case 5), multifocal positive signals were detected in the necrotic focus in the liver (Figures 2H,I). Intravascular bacteria detected within the heart of a bird (case 2) did not show positive hybridization signals. The bird which tested negative for *S. ornithocola* on bacteriological examination (case 6), did not show any positive signals throughout all tissues examined, including lung (Figures 1D–F), trachea and liver. Neither granulomatous lesions within the lung (Figures 1H,I) nor the bacterial structures within lung (Figures 1J–L) and necrotic foci of hepatic tissue (Figures 2K,L) of the cattle egret (case 7) produced positive hybridization signals. Application of hybridization buffer did not result in any signals (Figures 1C,F,I, 2C,F,I,L; Supplementary Figure 1C).

## Discussion

The present results show that *S. ornithocola* was associated with the spontaneous death of tits originating from the Arnsberg area, Germany. All animals examined (Table 1) were male birds. Except for the included cattle egret, all were members of the family *Paridae*, and found dead around spring, which is in accordance with previous publications (1–5, 20). Moreover, in agreement with previous reports, birds were found dead or in a state of severely reduced general condition (1, 2, 4, 5).

Necrotizing pneumonia together with other pulmonary lesions represented the most prominent histological finding in animals testing positive for *S. ornithocola* in bacterial culture and PCR. Considering the predominant lung lesions together with the observed lesion in the trachea of one bird, an aerogenic route of transmission with possible subsequent hematogenous spread seems very likely. Intralesional bacteria in lung, trachea and liver revealed positive hybridization signals (Figures 1, 2) using the antisense DNA probe specifically designed to detect *S. ornithocola*. Interestingly, one animal lacking obvious histological lung lesions in addition to having conspicuous intravascular bacterial structures in the lung tested positive for the bacterium by culture and PCR. Using *in situ* hybridization, the intravascular bacteria were identified as *S. ornithocola*. The presence of the bacterium within vascular lumina could also partly explain positive results in culture and PCR, despite the lack of histologically observable lesions (1). The isolated intravascular hybridization signal combined with the positive culture and PCR results could indicate hematogenous spread of the bacterium that led to fatal sepsis even before morphologic lesions became apparent. Unfortunately, the exact significance of the *in situ*-negative intravascular bacteria in a cardiac vessel of one animal could not be conclusively clarified. A great tit lacking obvious pulmonary lesions that tested negative for the bacterium in culture was included as negative control to exclude false-positive signals in inconspicuous avian lung tissue.

In addition, a cattle egret with heterophilic to necrotizing and partially granulomatous changes within lung and liver due to microbiologically confirmed infection with another member of the phylum Pseudomonadota (*Salmonella* ser. Typhimurium) was included to rule out unspecific signals caused by incidental gram-negative bacteria. The DNA probe designed for this study is not only the first CISH probe detecting *S. ornithocola* published, but it also allows, for the first time, a spatial co-localization of the suspected pathogen with histological lesions. Five tits tested positive for *S. ornithocola* in bacteriology (cases 1–5), three of them (cases 1, 2, 5) showing necrotizing pneumonia as previously described (1–3, 5). Positive hybridization signals were detected within necrotizing pulmonary lesions. Moreover, in birds lacking histological lesions within lung tissue, positive hybridization signals were found in a pulmonary vascular lumen (case 3) and a necrotizing lesion within the trachea (case 4). No positive precipitates were found in the negative controls that tested negative in bacteriological examination nor after treatment with hybridization buffer only. This suggests a high specificity and sensitivity of the developed probe, especially in animals with obvious histological lesions (14).

Although Koch's postulates are thus not completely fulfilled, a positive CISH signal within lesions provides further evidence of a possible causal relationship between infection with *S. ornithocola* and the emergence of a necrotizing pneumonia that represents the suspected cause of death in five of the deceased tits, included within this study. As noted by Peniche et al. (4), other lesions not considered classically associated with *S. ornithocola* infection might now be brought into a causal relationship with the bacterium, such as tracheitis and hepatocellular necrosis as observed in the present study. The multifocal distribution of bacteria as well as the detection of intravascular CISH-signals could indicate that *S. ornithocola* is able to cause systemic disease with lesions other than pneumonia or even fatal sepsis. Further investigations might shed light on the organ spectrum affected by this bacterium and possibly reveal new insights into its pathogenesis. The newly developed CISH probe may also be used to retrospectively examine archived material from tits and related birds with similar lesions to determine whether *Suttonella*-associated deaths have already occurred previously but have gone unnoticed. In addition, it can be used to screen for *S. ornithocola* not only in tits but in other bird populations including ornamental birds to determine how widespread this bacterium is in birds and whether there might be silent carriers and reservoirs.

## Data availability statement

Publicly available datasets were analyzed in this study. This data can be found at: GenBank: AJ717394.1.

## Ethics statement

The animals examined were wild birds found dead and examined for diagnostic purposes.

## Author contributions

MP, WB, and PW: conceptualization. EL and PW: methodology. EL, MP, and SM: investigation. EL: visualization. EL, WB, and PW: writing—original draft. EL, MP, SM, WB, and PW: writing—review and editing. All authors contributed to the article and approved the submitted version.

## Funding

This Open Access publication was funded by the Deutsche Forschungsgemeinschaft (DFG, German Research Foundation) − 491094227 Open Access Publication Costs and the University of Veterinary Medicine Hannover Foundation.

## Conflict of interest

Authors MP and SM were employed by Chemical and Veterinary Investigation Office Westphalia. The remaining authors declare that the research was conducted in the absence of any commercial or financial relationships that could be construed as a potential conflict of interest.

## Publisher's note

All claims expressed in this article are solely those of the authors and do not necessarily represent those of their affiliated organizations, or those of the publisher, the editors and the reviewers. Any product that may be evaluated in this article, or claim that may be made by its manufacturer, is not guaranteed or endorsed by the publisher.
