# Supplementary material for: Suttonella ornithocola detected within lesions of tit birds (Paridae) from epidemic death episodes in Germany, 2018–2020
Source: Front Vet Sci. 2022 Sep 8;9:977570. doi: 10.3389/fvets.2022.977570 (PMC9493112; doi:10.3389/fvets.2022.977570)
Supplement: Supplementary file 1 [file Data_Sheet_1.docx]

Supplementary Material

# Supplementary Figures

#
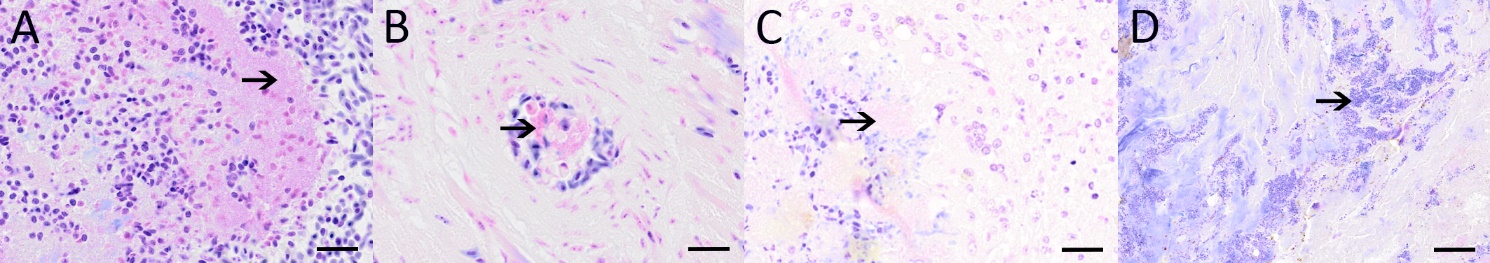


**Supplementary Figure 1:** Gram-stain of bacterial structures (arrows) reveals the presence of gram-negative bacteria. Gram-stain was performed on sections of the lung of a tit showing necrotizing pneumonia (A; case 2) and a cardiac vessel of the same animal (B; case 2) as well as within the liver of the cattle egret (C; case 7). Positive control (D) shows gram-positive coccoid bacteria (arrow) in bovine dermatitis. Bars: 20 µm.


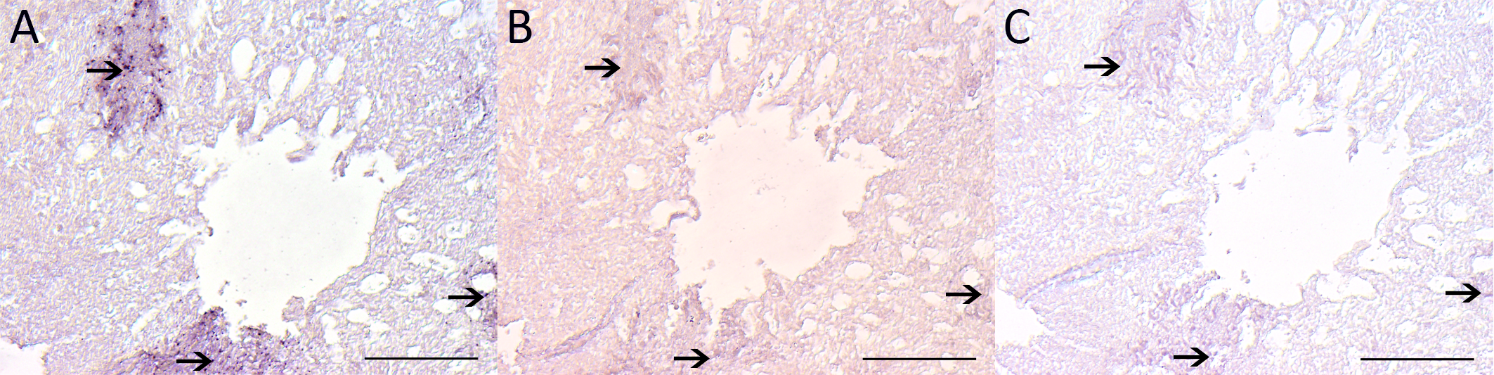


**Supplementary Figure 2:** Pulmonary lesions within an affected tit (case 5; A-C) characterized by moderate loss of organotypical architecture with accumulation of cellular debris and inflammatory cells as well as intralesional bacterial structures. Pictures illustrate hybridization results after incubation with the antisense (A) and sense (B) probes and with hybridization buffer only (C). Robust specific signals (A; purple precipitate; arrows) were only detected after using the antisense probe. The respective areas of interest are correspondingly marked with arrows in picture B and C. Pictures displaying *in situ* hybridization were taken using differential interference contrast (Nomarski) microscopy. Bars: 100 µm.
